# PPARγ and LXR Signaling Inhibit Dendritic Cell-Mediated HIV-1 Capture and *trans*-Infection

**DOI:** 10.1371/journal.ppat.1000981

**Published:** 2010-07-01

**Authors:** Timothy M. Hanley, Wendy Blay Puryear, Suryaram Gummuluru, Gregory A. Viglianti

**Affiliations:** Department of Microbiology, Boston University School of Medicine, Boston, Massachusetts, United States of America; NIH/NIAID, United States of America

## Abstract

Dendritic cells (DCs) contribute to human immunodeficiency virus type 1 (HIV-1) transmission and dissemination by capturing and transporting infectious virus from the mucosa to draining lymph nodes, and transferring these virus particles to CD4+ T cells with high efficiency. Toll-like receptor (TLR)-induced maturation of DCs enhances their ability to mediate *trans*-infection of T cells and their ability to migrate from the site of infection. Because TLR-induced maturation can be inhibited by nuclear receptor (NR) signaling, we hypothesized that ligand-activated NRs could repress DC-mediated HIV-1 transmission and dissemination. Here, we show that ligands for peroxisome proliferator-activated receptor gamma (PPARγ) and liver X receptor (LXR) prevented proinflammatory cytokine production by DCs and inhibited DC migration in response to the chemokine CCL21 by preventing the TLR-induced upregulation of CCR7. Importantly, PPARγ and LXR signaling inhibited both immature and mature DC-mediated *trans*-infection by preventing the capture of HIV-1 by DCs independent of the viral envelope glycoprotein. PPARγ and LXR signaling induced cholesterol efflux from DCs and led to a decrease in DC-associated cholesterol, which has previously been shown to be required for DC capture of HIV-1. Finally, both cholesterol repletion and the targeted knockdown of the cholesterol transport protein ATP-binding cassette A1 (ABCA1) restored the ability of NR ligand treated cells to capture HIV-1 and transfer it to T cells. Our results suggest that PPARγ and LXR signaling up-regulate ABCA1-mediated cholesterol efflux from DCs and that this accounts for the decreased ability of DCs to capture HIV-1. The ability of NR ligands to repress DC mediated *trans*-infection, inflammation, and DC migration underscores their potential therapeutic value in inhibiting HIV-1 mucosal transmission.

## Author Summary

Heterosexual transmission is the primary mode of HIV transmission worldwide. In the absence of an effective vaccine, there is an increasing demand for the development of effective microbicides that block HIV sexual transmission. Dendritic cells (DCs) play a critical role in HIV transmission by efficiently binding virus particles, migrating to lymph nodes, and transmitting them to CD4+ T cells, a process called *trans*-infection. In addition, DCs secrete proinflammatory cytokines that create a favorable environment for virus replication. DC maturation by pathogen-encoded TLR ligands or proinflammatory cytokines dramatically increases their capacity to capture HIV, migrate to lymphoid tissue, and *trans*-infect T cells. Here, we report that signaling through the nuclear receptors PPARγ and LXR prevents DC maturation and proinflammatory cytokine production, as well as migration. In addition, PPARγ and LXR signaling prevents efficient DC capture and transfer of infectious HIV by increasing ABCA1-mediated cholesterol efflux. Our studies suggest that PPARγ and LXR may be targets for drugs that can inhibit specific aspects of HIV mucosal transmission, namely inflammation, migration, and virus capture and transfer. These findings provide a rationale for considering PPARγ and LXR agonists as potential combination therapies with conventional anti-viral microbicides that target other aspects of mucosal HIV transmission.

## Introduction

Worldwide, heterosexual transmission accounts for most new HIV-1 infections, with a majority of these occurring in developing countries [1], [2]. Clearly, controlling heterosexual transmission of HIV-1 would be a significant step toward reducing this global pandemic. To achieve this goal, it will be important to delineate the cellular and molecular events that promote or restrict virus transmission and dissemination.

Immune cells within the vaginal, cervical, or rectal mucosa are thought to be the primary targets of infection in the sexual transmission of HIV-1 [1], [3], [4]. These target cells include sub-epithelial CD4+ T lymphocytes, intra-epithelial Langerhans cells, macrophages, submucosal plasmacytoid DCs (pDCs), and myeloid (or conventional) DCs (mDCs) located within the lamina propria [4], [5], [6], [7], [8], [9], [10], [11]. DCs, in particular, play a central role in HIV-1 transmission. DCs are thought to capture cell-free HIV-1 particles from the intralumenal space or from the mucosa after transcytosis across or leakage of HIV-1 particles through the epithelial barrier or by contacting HIV-1-infected cells introduced into the mucosa through abrasions or ulcerative lesions [6], [12], [13]. In addition, studies examining vaginal transmission of SIV_mac_ in a rhesus macaque model of AIDS have implicated DCs in virus dissemination from the mucosa to draining lymph nodes [6], [14]. Moreover, DCs are the predominant infected migratory cell type harboring HIV-1 from virus exposed cervical tissue explants [15] supporting the idea that they are involved in virus dissemination. Upon capture, DCs can deliver infectious HIV-1particles to draining lymph nodes that contain large numbers of CD4+ T cells [16], [17]. The close contact between virus-laden DCs and CD4+ T cells facilitates cell-to-cell transmission and viral spread [18], [19]. In addition to their roles in virus transmission and dissemination, DCs can produce proinflammatory cytokines that create a microenvironment that favors virus replication [20], [21], [22]. Recent reports have demonstrated that DCs matured by exposure to pathogens encoding Toll-like receptor (TLR) ligands or to proinflammatory cytokines are capable of enhanced HIV-1 *trans*-infection [23], [24], [25] and chemokine-directed migration [26], [27], suggesting that agents capable of preventing inflammation and DC maturation may be able to limit HIV-1 transmission and dissemination.

NRs are a superfamily of ligand-activated transcription factors that includes classic hormone receptors, as well as the so-called orphan receptors and adopted orphan receptors whose natural ligands are either unknown or recently discovered [28], [29]. Included in these latter two families are peroxisome-proliferator activated receptors (PPAR) and liver X receptors (LXR). Ligand-activated PPARγ and LXR are bifunctional modulators of gene expression, capable of either activating or repressing transcription in a promoter-specific manner. Importantly, PPARγ and LXR are potent inhibitors of inflammation and are capable of repressing cytokine and chemokine production by Toll-like receptor (TLR)-activated macrophages and DCs through *trans*-repression mechanisms involving the failure to clear co-repressor complexes from promoters or through direct antagonism of transcription factors such as the p65 subunit of NF-κB, AP-1, STATs, and IRF3 [30], [31], [32], [33], [34], [35], [36], [37], [38]. The effects of PPARγ and LXR on TLR signaling are complex and a number of studies have demonstrated that each NR inhibits different subsets of inflammatory genes [32], [34]. For example, LXR signaling represses TLR4-induced expression of iNOS, COX-2, and IL-6 in murine macrophages, while PPARγ signaling represses IL-1β, GCSF, MCP-1, MCP-3, and MIP-1α expression [32]. Here, we show that PPARγ and LXR signaling acutely prevents TLR-activated expression of the proinflammatory cytokines TNF-α, IL-6, and IL-8, which have been implicated as co-factors for enhanced mucosal transmission of HIV-1. Moreover, PPARγ and LXR signaling inhibit the expression of the chemokine receptor CCR7, thereby preventing DC chemotaxis in response to gradients of CCL21, a process thought to be involved in DC migration from mucosal surfaces to draining lymph nodes.

As opposed to their inhibitory effects on inflammatory gene expression, ligand-activated PPARγ and LXR induce expression of genes involved in lipid and cholesterol metabolism, as well as cholesterol transport, including ABCA1 and ABCG1 [29], [39], [40], [41]. Importantly, many studies have demonstrated that cholesterol plays an essential role in HIV-1 biology. Cholesterol must be present in both the target cell membranes and HIV-1 particles for efficient virus binding and fusion [42], [43], [44], [45], [46]. In addition, nascent HIV-1 particles bud through cholesterol-rich lipid rafts [47], [48] and infectious particles enter target cells through cholesterol-rich lipid rafts [42], [49], [50]. Finally, studies using the cholesterol chelator, methyl-β-cyclodextrin, demonstrated that cholesterol is required for DC binding of virus particles [51]. Interestingly, PPARs and LXR are expressed at high levels in HIV-1 target cells such as macrophages and DCs [28], [29]. Therefore, we hypothesized that PPARγ and LXR-mediated changes in cholesterol metabolism and trafficking might contribute to their ability to inhibit the transmission of HIV-1 from DCs to T cells. Our results demonstrate that PPARγ and LXR signaling inhibit the capture of HIV-1 by DCs, and its subsequent transfer to CD4+ T cells. These effects are due to up-regulation of ABCA1-dependent cholesterol efflux, a mechanism distinct from the effects of PPARγ and LXR signaling on DC migration and proinflammatory cytokine production. Collectively, our data suggest that the bifunctional activities of ligand activated PPARγ and LXR can be exploited to inhibit multiple distinct steps in HIV-1 mucosal transmission and dissemination.

## Results

### Treatment with PPARγ and LXR ligands prevents the maturation of MDDCs

**Figure 1:**
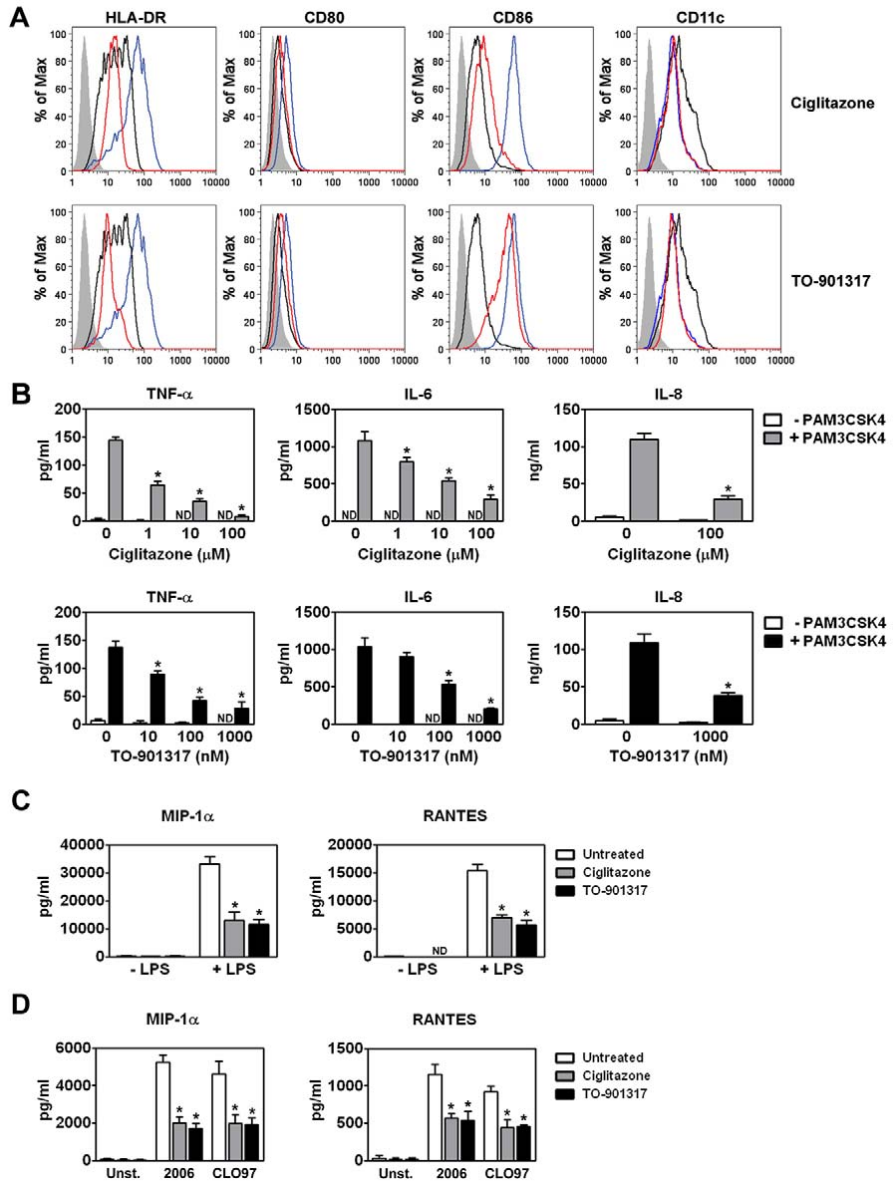
PPARγ and LXR ligand treatment prevents MDDC maturation and pro-inflammatory cytokine production. (A) MDDCs were treated with LPS for 48 hours in the presence or absence of the PPARγ ligand ciglitazone (100 µM; upper panel) or the LXR ligand TO-901317 (1 µM; lower panel). NR ligands were added one hour prior to TLR ligand addition. MDDCs were then stained for surface expression of HLA-DR, CD80, CD86, and CD11c and analyzed by flow cytometry. Shaded histogram, isotype control. Black line, iMDDCs. Blue line, LPS-treated MDDCs. Red line, LPS and nuclear receptor ligand-treated MDDCs. (B) MDDCs were treated with PAM3CSK4 in the presence or absence of 100 µM ciglitazone (upper panels) or 1 µM TO-901317 (lower panels). Cell-free supernatants were analyzed for TNF-α, IL-6, and IL-8 by ELISA. (n = 3) (C) MDDCs were treated with 100 ng/ml LPS in the presence or absence of 100 µM ciglitazone or 1 µM TO-901317. Cell-free supernatants were analyzed for MIP-1α and RANTES. (n = 3) (D) pDCs were treated with 5 µM CpG ODN 2006 or 1 µg/ml CLO97 in the presence or absence of 100 µM ciglitazone or 1 µM TO-901317. Cell-free supernatants were analyzed for MIP-1α and RANTES. (n = 4). * p<0.001 compared to TLR ligand-treated controls; ND, not detected.

TLR signaling induced by sexually transmitted pathogens is thought to enhance HIV-1 mucosal transmission in part by promoting local inflammation. Inflammation not only activates HIV-1 target cells but, importantly, it also induces DC maturation and the subsequent migration of HIV-1-carrying DCs to local lymph nodes where they can contribute to virus dissemination [16], [17]. We were therefore interested in determining whether the anti-inflammatory activities of ligand-activated PPARγ and LXR [34], [52], [53] could be exploited to limit DC functions involved in HIV-1 transmission and pathogenesis. To examine the effects of PPARγ and LXR signaling on DC maturation, human monocyte-derived DCs (MDDCs) were treated with *E. coli* K12 LPS, a TLR4 ligand, in the presence or absence of ligands for PPARγ and LXR. As expected, LPS treatment upregulated the expression of surface markers associated with maturation, such as HLA-DR, CD80, CD86, and CD83, downregulated the expression of surface markers associated with an immature phenotype, such as the C-type lectin DC-SIGN, but had no effect on the expression of the pan-DC marker CD11c (Figure 1A and data not shown). Notably, treatment of MDDCs with the PPARγ ligand ciglitazone or the LXR ligand TO-901317 inhibited LPS-dependent upregulation of cell-surface expression of HLA-DR, CD80, and CD86 (Figure 1A). Similarly, we found that ciglitazone or TO-901317 treatment inhibited human MDDC maturation in response to the TLR2 ligand PAM3CSK4 (data not shown).

We next examined the effects of ciglitazone and TO-901317 treatment on TLR-induced proinflammatory cytokine and chemokine production. We found that treatment with these PPARγ and LXR ligands prevented the release of proinflammatory cytokines and chemokines such as TNF-α, IL-6, and IL-8 by PAM3CSK4-activated MDDCs (Figure 1B). In addition, PPARγ and LXR treatment also prevented the release of the chemokines MIP-1α and RANTES, which are important for the recruitment of CD4+ T cells to sites of infection, both from MDDC in response to the TLR4 ligand LPS (Figure 1C) and from plasmacytoid DCs (pDCs) in response to the TLR7 ligand CLO97 and the TLR9 ligand CpG ODN 2006 (Figure 1D). Importantly, PPARγ and LXR signaling inhibited TLR-induced proinflammatory cytokine and chemokine production coincident with TLR ligation (data not shown), suggesting that NR-mediated inhibition most likely acts through a *trans*-repression mechanism [34]. The concentrations of the PPARγ ligand ciglitazone and the LXR ligand TO-901317 necessary to see a reduction in DC maturation and the production of pro-inflammatory cytokines and chemokines did not affect MDDC viability as measured by LDH release or mitochondrial activity (Figure S1 and data not shown).

### NR signaling prevents MDDC migration in response to CCL21

**Figure 2:**
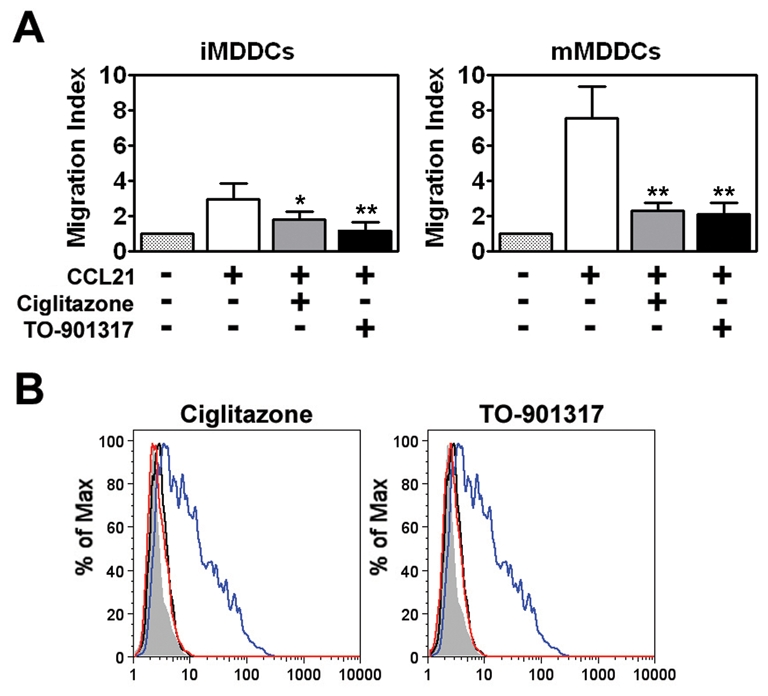
PPARγ and LXR ligand treatment prevents MDDC migration in response to CCL21. (A) Immature (left panel) or mature (right panel) MDDCs were seeded in the upper chamber of a Transwell insert and allowed to migrate in response to medium or a CCL21 gradient in the lower chamber. Migration indexes were determined after four hours. On average, approximately 14% of immature MDDCs (0.34×10^5^ cells) and 40% of LPS-matured MDDCs (1.08×10^5^ cells) migrated through the Transwell insert in response to CCL21. (n = 3) * p<0.005, ** p<0.001 compared to CCL21-treated controls (B) MDDCs were treated with LPS for 48 hours in the presence or absence of 100 µM ciglitazone or 1 µM TO-901317 and analyzed for CCR7 expression by flow cytometry. Shaded histogram, isotype control. Black line, iMDDCs. Blue line, LPS-treated MDDCs. Red line, LPS and nuclear receptor ligand-treated MDDCs.

In addition to transmitting HIV-1 to T cells with high efficiency, DCs can also contribute to HIV-1 pathogenesis by binding virus and then migrating from mucosal sites of infection to regional lymph nodes. In this way, DCs can contribute to viral dissemination. Studies have shown that mature DCs have a greater migratory capacity than immature DCs [26], [27]. This led us to examine whether NR signaling would also inhibit MDDC migration through a 5 µm pore size Transwell insert in response to the chemokine CCL21, which has been shown to be important for DC migration *in vivo* [27]. We found that LPS-matured MDDCs (mMDDCs) migrated in response to a CCL21 gradient and that co-treatment with PPARγ or LXR ligands repressed this migration approximately 2-fold (Figure 2A). In contrast, immature MDDCs (iMDDCs) migrated quite poorly in response to CCL21 and, consequently, NR ligand treatment had a limited effect. Expression of CCR7, a receptor for CCL21, is upregulated in DCs in response to TLR engagement [26], [54]. Notably, treatment with PPARγ and LXR ligands prevented the LPS-induced upregulation of CCR7 (Figure 2B), which may partly explain why NR ligand-treated MDDCs migrate poorly in response to CCL21. Together, these data suggest that PPARγ and LXR signaling inhibit DC migration by preventing TLR-induced DC maturation.

### NR ligands inhibit MDDC-mediated *trans*-infection of HIV-1 to T cells

**Figure 3:**
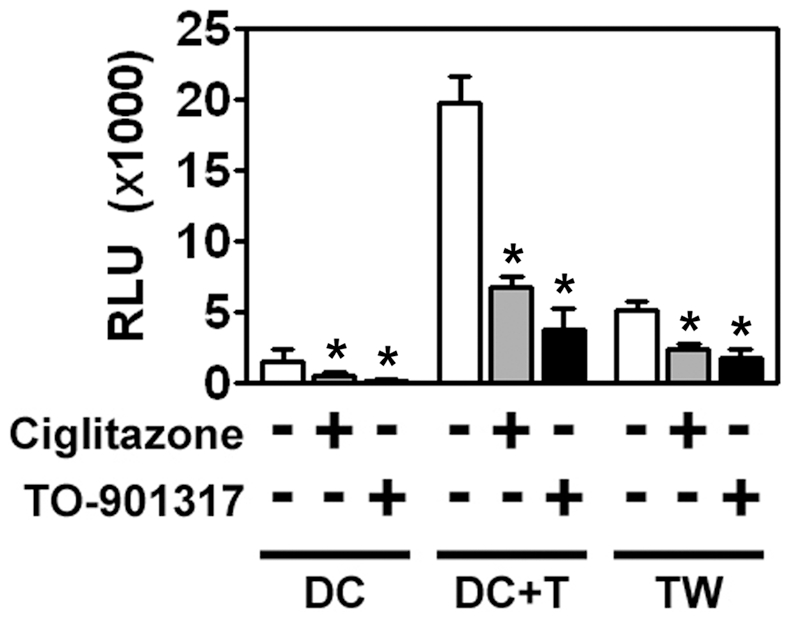
MDDCs are poorly infected with HIV-1 but can mediate *trans*-infection of T cells. iMDDCs were treated for 48 hours with 100 µM ciglitazone or 1 µM TO-901317, washed extensively, and then incubated with a single round replication-competent HIV-1 reporter virus encoding luciferase and pseudotyped with R5-tropic envelope glycoproteins. After four hours, unbound virus was removed by extensive washing and the MDDCs were incubated directly with PM1 T cells or separated from the PM1 T cells by a Transwell insert with a 0.4 µm membrane for 48 hours. The cells were then harvested, lysed, and assayed for luciferase activity. (n = 4) DC, MDDCs cultured in the absence of T cells. DC + T, MDDCs cultured directly with T cells. TW, MDDCs separated from T cells by a Transwell insert. * p<0.001.

DCs are thought to play a critical role in virus dissemination by capturing HIV-1 and transferring it to T cells [5], [24], [55]. We therefore examined whether NR ligands could modulate DC-mediated HIV-1 *trans*-infection. iMDDCs were treated with ciglitazone or TO-901317 for 48 hours, extensively washed, and then incubated for four hours with either a single-round replication-competent HIV-1 reporter virus packaged with an R5-tropic envelope or with wild-type HIV-1. Following incubation with the virus, MDDCs were washed extensively to remove unbound virus and then cultured directly with autologous T cells or in the upper well of a Transwell insert separated from the T cells by a 0.4 µm membrane. Although HIV-1 replicated very poorly in immature MDDCs (Figure 3), we found that DCs were able to mediate T cell infection when directly cultured with the T cells or when separated from them by the Transwell insert (Figure 3), suggesting that a portion of the MDDC-mediated *trans*-infection is mediated by either exosome-associated HIV-1 [56] or virus shed from the surface of MDDCs [57].

**Figure 4:**
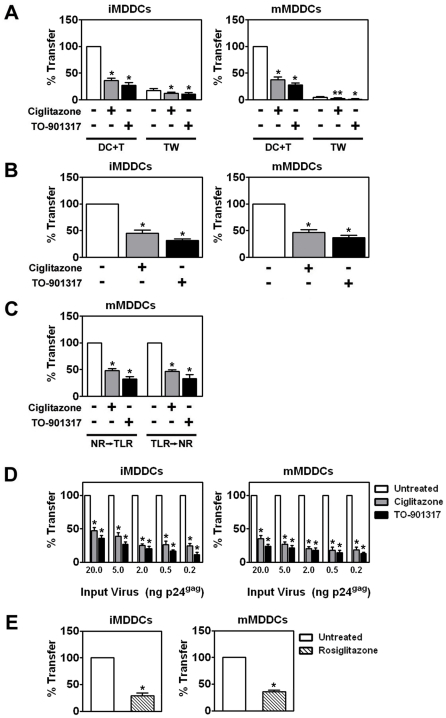
NR ligand treatment prevents HIV-1 *trans*-infection. (A) Immature (left panel) and LPS-matured (right panel) MDDCs were treated for 48 hours with 100 µM ciglitazone or 1 µM TO-901317 (NR ligands were added one hour prior to TLR ligand addition), incubated with replication competent HIV-1_ADA_, washed, and cultured directly with autologous T cells (DC + T) or separated from the T cells by a Transwell insert (TW) for a period of 48 hours. Virus transfer and replication was measured by p24^gag^ ELISA. DC + T, MDDCs cultured directly with T cells. TW, MDDCs separated from T cells by a Transwell insert. Mean transfer/replication values: 9.18 ng p24^gag^ for untreated iMDDCs cultured directly with T cells; and 85.5 ng p24^gag^ for untreated mMDDCs cultured directly with T cells. (n = 5) * (B) Immature (left panel) and PAM3CSK4-matured (right panel) MDDCs were treated for 48 hours with 100 µM ciglitazone or 1 µM TO-901317, washed extensively, and then incubated with HIV-1_ADA_. After four hours, unbound virus was removed by extensive washing and the MDDCs were incubated directly with autologous primary T for 48 hours. The cells were then harvested, lysed, and assayed for virus transfer by p24^gag^ ELISA. (n = 3) (C) MDDCs were treated with LPS and either 100 µM ciglitazone or 1 µM TO-901317 for 48 hours (NR → TLR) or with LPS for 48 hours prior to treatment with NR ligands for an additional 48 hours (TLR → NR). MDDCs were incubated with HIV-1_ADA_, washed, and cultured directly with autologous T cells. Virus transfer was measured by p24^gag^ ELISA. (n = 3) (D) Immature (left panel) and LPS-matured (right panel) MDDCs were treated for 48 hours with 100 µM ciglitazone or 1 µM TO-901317 and incubated with increasing amounts of HIV-1_ADA_, washed extensively, and then cultured with autologous T cells. Virus transfer was measured by p24^gag^ ELISA. (n = 3) (E) Immature (left panel) and LPS-matured (right panel) MDDCs were treated for 48 hours with 1 µM rosiglitazone (hatched bars), incubated with HIV-1_ADA_, washed, and cultured directly with autologous T cells. Virus transfer was measured by p24^gag^ ELISA. (n = 3) * p<0.001, ** p<0.005 compared to untreated controls.

Most importantly, we found that PPARγ and LXR ligands inhibited *trans*-infection up to 5-fold underscoring their potential to limit HIV-1 transmission (Figure 3). NR signaling inhibits *trans*-infection of T cells by both single-round replication competent virus (Figure 3) and wild-type replication competent virus (Figure 4A), suggesting that the majority of virus transferred to T cells is due to virus captured by the DC and not due to newly synthesized virus. Because mature DCs capture and transfer HIV-1 to T cells with higher efficiency than immature DCs [23], [24], [25], we next determined whether PPARγ or LXR ligands could inhibit *trans*-infection mediated by LPS- or PAM3CSK4-matured MDDCs. PPARγ and LXR signaling repressed *trans*-infection of autologous primary T cells mediated by both immature, LPS-matured MDDCs (Figure 4A), and PAM3CSK4-matured MDDCs (Figure 4B), suggesting that the repression is independent of MDDC maturation. To confirm NR-dependent maturation-independent repression of DC-mediated HIV-1 trans-infection, we matured MDDCs with LPS for two days prior to treatment with NR ligands and then assayed for HIV-1 transfer. As seen in figure 4C, the ability of mature MDDCs to transfer virus was impaired when treated with PPARγ and LXR ligands. In addition, we found that PPARγ and LXR ligand treatment of MDDCs prevented trans-infection over a wide range of input virus (Figure 4D). Of note, NR-ligand treatment inhibited immature and mature MDDC-mediated *trans*-infection of both R5- and X4-tropic envelope glycoprotein-pseudotyped single-round replication competent reporter viruses and replication-competent R5- and X4- tropic wild-type HIV-1 (data not shown). Together these data suggest that, unlike PPARγ- and LXR-mediated inhibition of migration, the inhibition of *trans*-infection is independent of the maturation state of the DC. Importantly, MDDC-mediated *trans*-infection is also inhibited by rosiglitazone (Figure 4E), a PPARγ agonist that is currently licensed for the systemic treatment of type II diabetes.

### PPARγ and LXR ligands inhibit *trans*-infection at least in part by blocking HIV-1 capture by MDDCs

**Figure 5:**
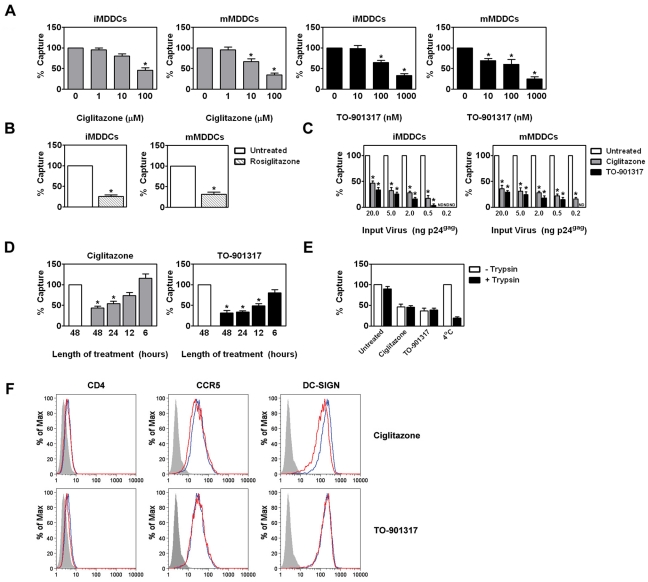
NR ligand treatment prevents HIV-1 *trans*-infection by blocking the capture of HIV-1 particles by MDDCs. (A) Immature (left panel) and mature (right panel) MDDCs were treated with increasing concentrations of ciglitazone or TO-901317 for 48 hours. The MDDCs were then incubated with 5 ng p24^gag^ equivalent of HIV-1_ADA_ and virus capture was measured by p24^gag^ ELISA. Mean capture values: 622.93 pg p24^gag^ for untreated iMDDCs; and 1.80 ng p24^gag^ for untreated mMDDCs. (n = 4) (B) Immature (left panel) and mature (right panel) MDDCs were treated with 1 µM rosiglitazone (hatched bars) for 48 hours and analyzed as in (A). (n = 3) (C) Immature (left panel) and LPS-matured (right panel) MDDCs were treated for 48 hours with 100 µM ciglitazone or 1 µM TO-901317 and incubated with increasing amounts of HIV-1_ADA_, washed extensively, and lysed. Virus capture was measured by p24^gag^ ELISA. (n = 3) (D) iMDDCs were treated with 100 µM ciglitazone or 1 µM TO-901317 for various times, incubated with HIV-1_ADA_, and assayed for virus capture by p24^gag^ ELISA. (n = 3) (E) Immature MDDCs were treated with ciglitazone (100 µM) or TO-901317 (1 µM) for 48 hours, incubated with HIV-1_ADA_ for four hours at 37°C, and then washed to remove unbound virus. Some cells were incubated with virus at 4°C (as indicated). The cells were then treated with 0.5% trypsin for 5 minutes at 37°C to degrade surface-bound virus particles, washed twice in culture medium, and lysed as above. Virus capture was measured by p24^gag^ ELISA. (n = 3) (F) iMDDCs were treated with either 100 µM ciglitazone or 1 µM TO-901317 for 48 hours and cell-surface expression of CD4, CCR5, and DC-SIGN was measured by flow cytometry. Shaded histogram, isotype control. Blue line, iMDDCs. Red line, nuclear receptor ligand-treated iMDDCs. * p<0.001 compared to untreated controls, ND, not detected.

Next, we wanted to examine the mechanism accounting for the inhibition of *trans*-infection. We began by examining the effects of PPARγ or LXR ligand treatment on HIV-1 binding to MDDCs. Ciglitazone and TO-901317 treatment led to a 2 to 5-fold decrease in the amount of HIV-1 associated with MDDCs as measured by an ELISA for the HIV-1 p24 capsid protein (Figure 5A). Another PPARγ ligand, rosiglitazone, was also tested and had a comparable effect on HIV-1 capture (Figure 5B). Treatment with these NR ligands also inhibited the capture of HIV-1 by DCs at 4°C, suggesting that NR ligand treatment prevents DC binding of HIV-1 (Figure S2). In addition, we found that PPARγ and LXR ligand treatment of MDDCs prevented capture over a wide range of input virus (Figure 5C). Although NR signaling can repress inflammatory gene expression by a *trans*-repression mechanism [30], [31], [32], [33], [34], [36], [37], [38], [52], it likely decreases HIV-1 capture through a different mechanism. MDDCs must be treated with PPARγ and LXR ligands for at least 12 hours in order to observe inhibition of virus capture (Figure 5D), suggesting that changes in cellular gene expression are required for the observed effect. Though the amount of virus captured by MDDCs upon NR ligand treatment was reduced, the relative amount of virus particles internalized was similar (Figure 5E) suggesting that reduced ability of MDDCs to capture HIV-1 particles upon NR ligand treatment was not due to gross reduction in cellular endocytic function. To confirm that NR ligand treatment does not alter the ability of MDDCs to internalize particles, we examined their effects on the ability of MDDCs to macropinocytose FITC-labeled dextran. NR ligand treatment had no effect on FITC-dextran internalization by immature or mature MDDCs (Figure S3 and data not shown).

### NR ligand treatment does not alter the expression of HIV-1 attachment factors

Our data suggest that changes in cellular gene expression are necessary for the observed decrease in HIV-1 capture by MDDCs. We therefore considered the possibility that PPARγ and LXR ligand treatment altered the expression of known HIV-1 attachment factors expressed on the surface of immature MDDCs. However, we found that NR ligand treatment did not alter the expression of CD4, CCR5, or DC-SIGN (Figure 5F), which have been implicated in DC capture of HIV-1 [5], [58]. Despite these findings, we cannot rule out whether NR signaling alters the expression of other factors implicated in HIV-1 attachment such as other C-type lectins [59], [60], [61], [62], heparan sulfate proteoglycans [63], [64], [65], or GSLs [66], [67], [68], [69], [70].

### NR ligand treatment prevents HIV-1 capture and transfer by myeloid DCs

**Figure 6:**
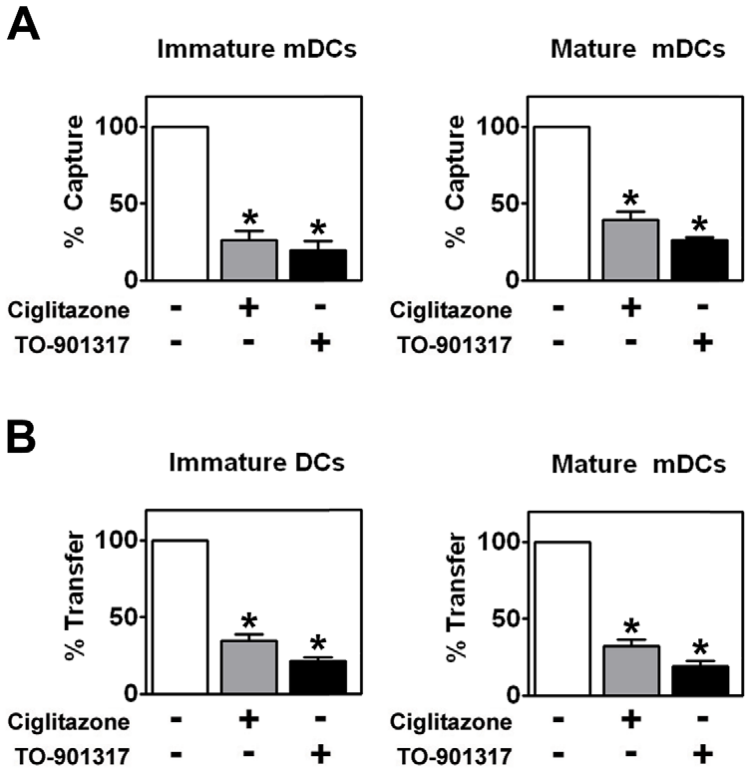
PPARγ and LXR ligand treatment inhibit myeloid DC-mediated HIV-1 capture and *trans*-infection. (A) Immature (left panel) and mature (right panel) mDCs were treated with 100 µM ciglitazone or 1 µM TO-901317 for 48 hours. The mDCs were then incubated with HIV-1_ADA_ and virus capture was measured by p24^gag^ ELISA. (n = 4) (B) Immature (left panel) and LPS-matured (right panel) mDCs were treated for 48 hours with 100 µM ciglitazone or 1 µM TO-901317, incubated with HIV-1_ADA_, washed, and cultured directly with autologous T cells. Virus transfer was measured by p24^gag^ ELISA. (n = 4) * p<0.001 compared to untreated controls.

Although MDDCs are a faithful representation of myeloid or conventional DCs (mDCs) with respect to their interactions with HIV-1 [25], we decided to utilize mDCs freshly isolated from the peripheral blood of healthy volunteers. We found that PPARγ and LXR signaling inhibited the ability of immature and LPS-matured mDCs to capture HIV-1_ADA_ and transfer it to autologous T cells (Figure 6) in a manner consistent with results obtained using MDDCs.

### PPARγ and LXR signaling does not prevent virological synapse formation

**Figure 7:**
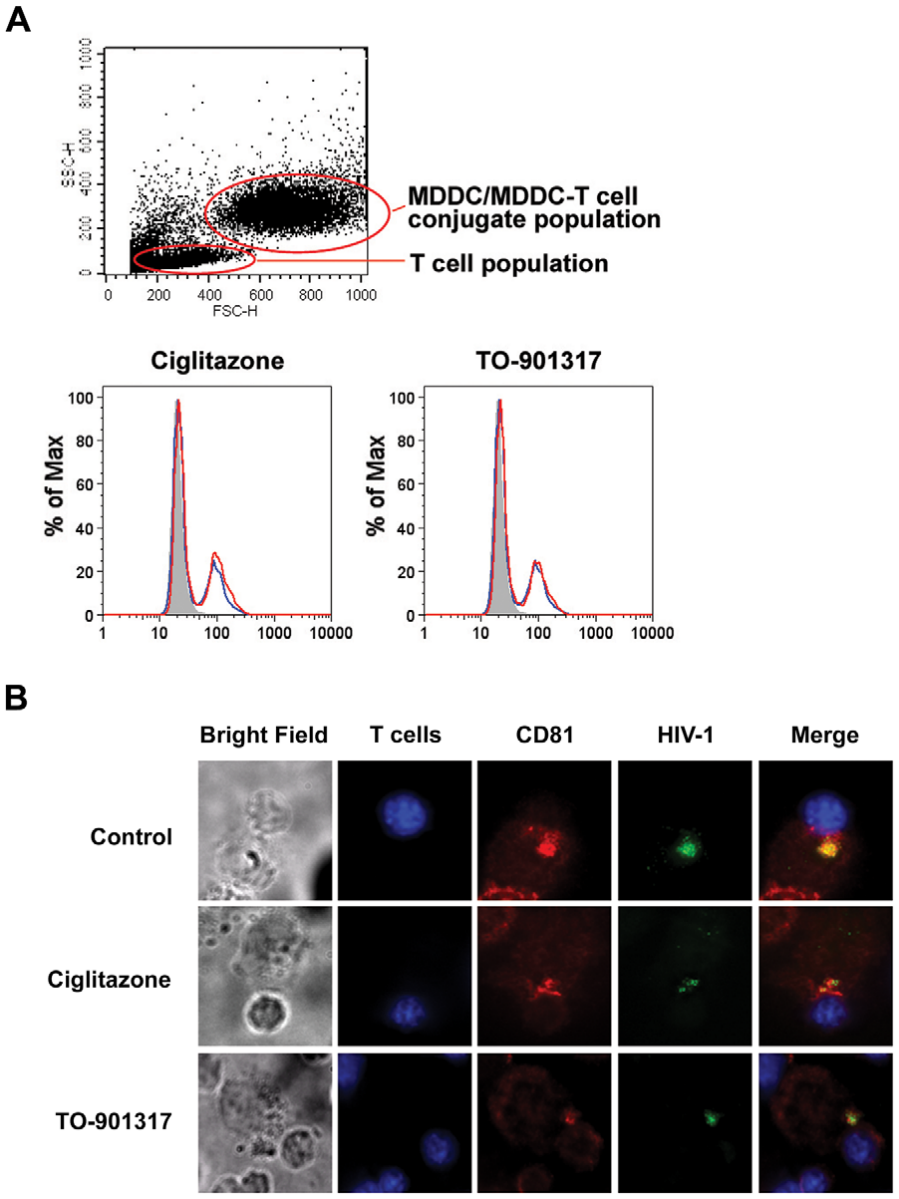
PPARγ and LXR ligand treatment do not prevent DC-T cell conjugate or virological synapse formation. (A) Primary autologous CD4+ T cells were labeled with the cytoplasmic dye CMTMR for 30 minutes at 37°C, washed three times with PBS to remove excess dye, and incubated overnight at 37°C. Following labeling, 5×10^5^ T cells were incubated with 2.5×10^5^ unlabeled iMDDCs for four hours at 37°C. The conjugates were then fixed in 2% paraformaldehyde and conjugate formation was assessed by flow cytometry with gating on the MDDC population. Shaded histogram, unlabeled MDDCs without T cells. Blue line, untreated MDDCs with T cells. Red line, nuclear receptor ligand-treated MDDCs with T cells. (B) Primary autologous CD4+ T cells were labeled with the cytoplasmic dye CMCA (CellTracker Blue, Molecular Probes) for 30 minutes at 37°C, washed three times with PBS to remove excess dye, and incubated overnight at 37°C. 2.5×10^5^ unlabeled LPS-matured MDDCs were incubated with 100 ng HIV-1_NL4-3_ virions packaged with Vpr-EGFP for four hours at 37°C, washed four times with PBS, and incubated with 5×10^5^ CMCA-labeled autologous T cells for four additional hours. The cells were fixed in 1% paraformaldehyde, stained with anti-CD81-PE, and analyzed by deconvolution microscopy.

Because direct DC-T cell contact is required for efficient virus transfer [24], [57] (and Figure 3), we wanted to determine whether NR ligand treatment interfered with the ability of MDDCs to form conjugates with T cells. Using a FACS-based conjugate formation assay [71], we determined that NR ligand-treated MDDCs were able to form conjugates with primary autologous T cells in a manner similar to untreated MDDCs (Figure 7A). Because NR ligand treatment did not alter the ability of DCs to form conjugates with T cells, we next wanted to examine whether such treatment prevented the formation of functional virological synapses between DCs and T cells. Confocal microscopy data suggest that PPARγ and LXR ligand-treated DCs are capable of forming virological synapses, as indicated by co-localization of virus and the tetraspanin CD81 at the site of DC-T cell contact (Figure 7B). However, number of virus particles localized at the virological synapse is decreased in NR ligand-treated cells. Taken together, our data suggest that NR signaling impairs the ability of MDDCs to transfer virus to T cells by inhibiting the capture of HIV-1 by MDDCs.

### PPARγ and LXR ligands inhibit HIV-1 capture by MDDCs in an envelope glycoprotein-independent manner

**Figure 8:**
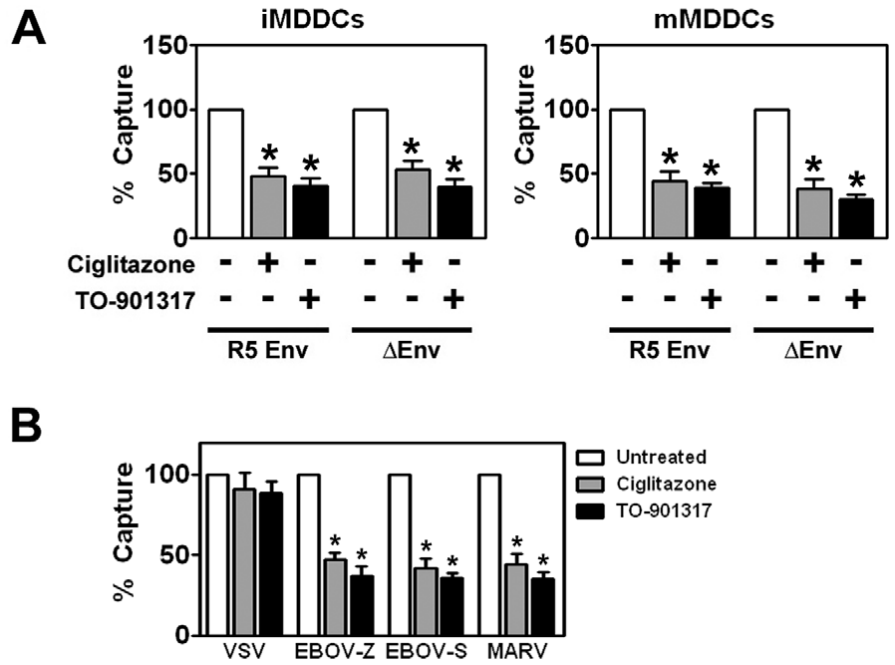
PPARγ and LXR ligand treatment prevents envelope glycoprotein-independent capture of virus by MDDCs. (A) Immature (left panel) and mature (right panel) MDDCs were treated with 100 µM ciglitazone or 1 µM TO-901317 and incubated with a single round replication-competent HIV-1 reporter virus encoding luciferase that was either pseudotyped with R5-tropic envelope glycoproteins (R5) or that lacked envelope glycoproteins (ΔEnv). Virus capture was measured by p24^gag^ ELISA. (n = 3) (B) Immature MDDCs were treated with100 µM ciglitazone or 1 µM TO-901317 and incubated with a single round replication-competent HIV-1 reporter virus encoding luciferase that was packaged with envelope glycoproteins from VSV (VSV-G), Ebola virus Zaire (EboV-Z), Ebola virus Sudan (EboV-S), or Marburg virus (MarV). Virus capture was measured by p24^gag^ ELISA. Mean capture values for untreated iMDDCs: 1.13 ng VSV-G-pseudotyped HIV-1; 251.83 pg EBOV-Z-pseudotyped HIV-1; 324.10 pg EBOV-S-pseudotyped HIV-1; and 415.07 pg MARV-pseudotyped HIV-1. (n = 3) * p<0.001 compared to untreated controls.

Recent studies have demonstrated that DCs can bind to infectious HIV-1 and envelope-deficient virus-like particles (VLPs) in a GSL-dependent, viral envelope glycoprotein-independent manner [72], [73]. We therefore wanted to assess whether triggering PPARγ and LXR signaling could alter the ability of MDDCs to bind virus independently of the envelope glycoprotein gp120. We found that PPARγ and LXR ligand treatment led to a 2 to 5-fold decrease in the amount of envelope glycoprotein (Env)-deficient HIV-1 particles captured by both immature and mature MDDCs (Figure 8A), suggesting that GSL-based virus-DC interactions may be targeted by NR signaling. To demonstrate that this interaction is truly envelope glycoprotein-independent, we also examined the effects of PPARγ and LXR signaling on the ability of DCs to capture HIV-1 particles pseudotyped with the glycoproteins of vesicular stomatitis virus (VSV), Ebola virus (EboV), and Marburg virus (MarV). As shown in figure 8B, treatment with PPARγ and LXR ligands inhibited the ability of DCs to capture EboV or MarV glycoprotein-pseudotyped HIV-1 particles, whereas the treatment had no effect on the ability of DCs to capture VSV-G-pseudotyped particles. Since, like HIV-1, both EboV and MarV glycoproteins are known to require cholesterol for infection [74], [75], whereas VSV-G does not [43], [75], [76], this suggested that PPARγ and LXR might be exerting their effects through the regulation of cellular cholesterol.

### PPARγ and LXR ligand inhibits HIV-1 capture by MDDCs in a cholesterol-dependent manner

**Figure 9:**
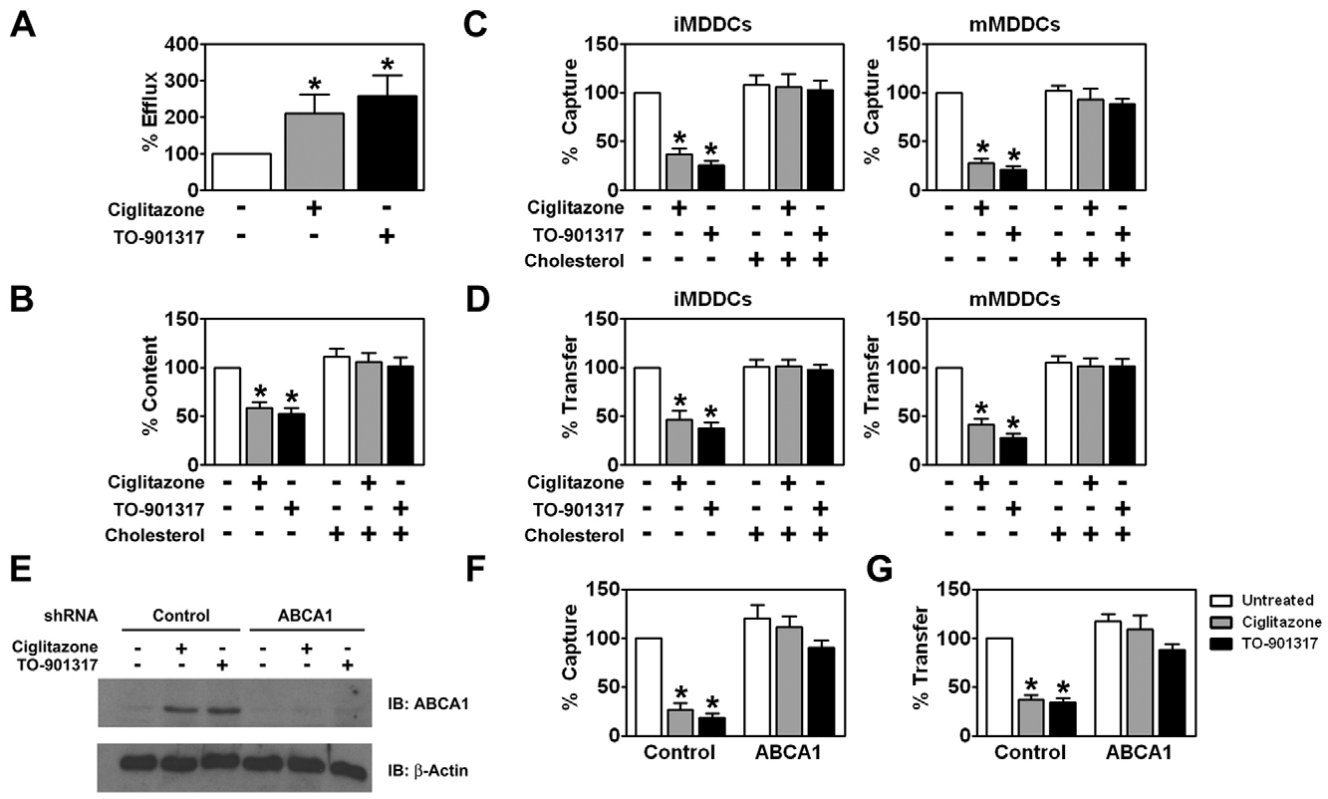
PPARγ and LXR signaling inhibit HIV-1 capture and transfer by MDDCs via ABCA1-dependent cholesterol efflux. (A) iMDDCs were treated for 48 hours with 100 µM ciglitazone or 1 µM TO-901317 and cholesterol efflux into the culture supernatant was measured. (n = 3) (B) iMDDCs were treated for 48 hours with 100 µM ciglitazone and 1 µM TO-901317. The cells were then lysed and cholesterol content of the cell lysates was measured. (n = 3) (C-D) Immature (left panel) and mature (right panel) MDDCs were treated with 100 µM ciglitazone or 1 µM TO-901317 for 48 hours. NR ligand-treated cells were then incubated with 300 µM cholesterol in the form of cholesterol-saturated methyl-β-cyclodextrin. Following cholesterol repletion, the cells were used in capture (C) and transfer assays (D) as described in the legend of figure 2. (n = 3) (E) iMDDCs transfected with either control shRNA or shRNA directed against ABCA1 were treated for 48 hours with 100 µM ciglitazone or 1 µM TO-901317. Levels of ABCA1 and β-actin were measured by immunoblot using an anti-ABCA1 and anti-β-actin antibodies, respectively. (n = 3) (F) iMDDCs transfected with either control shRNA or shRNA directed against ABCA1 were treated for 24 hours with 100 µM ciglitazone (gray bars) or 1 µM TO-901317 (black bars). The MDDCs were then incubated with HIV-1_ADA_ and virus capture was measured by p24^gag^ ELISA. (n = 3) (G) iMDDCs transfected with either control shRNA or shRNA directed against ABCA1 were treated for 24 hours with 100 µM ciglitazone (gray bars) or 1 µM TO-901317 (black bars). The MDDCs were then incubated with HIV-1_ADA_, washed extensively, and then incubated with autologous T cells. Virus transfer was measured by p24^gag^ ELISA. Transfer assays with shRNA-treated DCs were performed using cells from two independent donors. * p<0.001 compared to untreated controls.

Previous studies have shown that DC capture of HIV-1 is dependent upon the cholesterol content of the cell membrane [51]. Since both PPARγ and LXR are known to modulate genes involved in cholesterol metabolism and transport [29], [39], [40], [77], we were interested in determining whether ciglitazone or TO-901317 affected the cholesterol content of MDDCs. Treatment with PPARγ and LXR ligands increased cholesterol efflux from immature MDDCs approximately 2 to 3-fold (Figure 9A) and led to a concomitant 2-fold decrease in the amount of cholesterol in immature MDDCs (Figure 9B). We next wanted to see if cholesterol depletion resulting from PPARγ and LXR ligand treatment was responsible for the decreased ability of MDDCs to capture and transfer HIV-1. In order to do this, we replenished membrane cholesterol in PPARγ and LXR ligand-treated MDDCs using cholesterol-saturated methyl-β-cyclodextrin and assayed for HIV-1 capture and transfer to T cells. Cholesterol repletion of NR ligand-treated MDDCs with cholesterol-saturated methyl-β-cyclodextrin restored cholesterol content (Figure 9B) and, importantly, fully restored the ability of both immature and mature MDDCs to capture HIV-1 (Figure 9C) and transfer it to CD4+ T cells (Figure 9D).

PPARγ and LXR signaling upregulate expression of ATP-binding cassette protein A1 (ABCA1) that facilitates the apoA1-dependent efflux of cholesterol from cells [39], [41], [77]. We therefore examined whether treatment of DCs with ciglitazone or TO-901317 affected ABCA1 expression. We found by western blot analysis that both NR ligands increased ABCA1 expression (Figure 9E). Importantly, targeted knockdown of ABCA1 using shRNA abrogated the effect of PPARγ and LXR ligand treatment on cholesterol efflux (data not shown), HIV-1 capture by DCs (Figure 9F), and HIV-1 transfer to T cells (Figure 9G). These findings suggest that ligand-activated PPARγ and LXR mediate their effects through the depletion of cholesterol from the DC plasma membrane via the up-regulation of the ABCA1 cholesterol transport protein. It will be interesting to determine whether HIV-1 particles interact directly with cholesterol in the plasma membrane of DCs or with factors that localize to cholesterol-rich lipid rafts.

## Discussion

Sexual transmission of HIV-1 is enhanced by inflammatory and ulcerative co-infections with STI pathogens that cause diseases such as genital herpes, gonorrhea, syphilis, Chlamydia, bacterial vaginosis, and fungal infections [78], [79], [80], [81], [82], [83]. This enhanced susceptibility to infection may be due to a number of factors, including disruption of epithelial integrity [6], [11], [14], [84], [85], [86], [87], recruitment of HIV-1 target cells such as Langerhans cells, DCs, macrophages, and T lymphocytes to sites of inflammation [8], and activation of HIV-1 expression by pro-inflammatory cytokines or microbial components [21], [22], [88], [89], [90], [91], [92]. It is likely that STI pathogens enhance these latter two processes, at least in part, through engagement of the TLR family of innate immune receptors. Clearly, prophylactic methods that inhibit infection of the genital or rectal mucosa would significantly limit the global spread of HIV. To this end, considerable efforts have been directed toward the development of microbicides that interfere with virus integrity or with key steps in virus replication. However, to date, little attention has been paid to targeting cellular pathways involved in active suppression of inflammation and its effects on mucosal HIV-1 infection and virus dissemination. With this in mind, we have focused our efforts on the nuclear receptor family of transcription factors that have recently been shown to be potent inhibitors of TLR-induced inflammation [30], [31], [32], [33], [34], [36], [37], [38]. Here, we demonstrate that PPARγ and LXR signaling inhibit several aspects of DC biology that are important for HIV-1 mucosal transmission. These include TLR-induced pro-inflammatory cytokine expression, DC migration in response to the chemokine, CCL21, and, importantly, DC-mediated capture of infectious virus particles and *trans*-infection of CD4+ T cells. Our findings highlight the therapeutic potential of PPARγ and LXR ligands as topical treatments that could be used in conjunction with conventional microbicides to limit mucosal transmission of HIV-1.

DC-mediated *trans*-infection of T cells is thought to play a critical role in the mucosal transmission of HIV-1. Studies suggest that DCs can mediate *trans*-infection either by internalizing infectious virions into a protected tetraspanin-rich intracellular compartment, or deep membrane invaginations contiguous with the cell surface, and releasing them for the subsequent infection of T cells [5], [56], [73], [93], [94], [95] or by retaining virions at the cell surface and transferring them to T cells [57], [95]. Regardless of the mechanism, maturation of DCs with ligands for TLRs such as TLR4 and TLR2/TLR1 increases DC-mediated HIV-1 capture and *trans*-infection of T cells. DC maturation also contributes to HIV-1 mucosal transmission in a number of other ways. Mature DCs create a pro-inflammatory environment that favors virus replication [20], [88], [96], [97], [98] and leads to disruption of the mucosal integrity [83], [99]. Mature DCs may also contribute to virus dissemination by virtue of their enhanced ability to traffic to regional lymph nodes in response to chemokine gradients and, once there, transfer virus to resident CD4+ T cells. Here we show that PPARγ or LXR ligand treatment can prevent DC maturation as measured by the expression of cell surface markers such as HLA-DR, CD80, and CD86 (Figure 1A). Importantly, treatment with PPARγ or LXR ligands also potently inhibit expression of maturation-associated pro-inflammatory cytokines (Figure 1B), such as TNF-α and IL-6 and the pro-inflammatory chemokine, IL-8, that have been shown to augment HIV-1 replication in infected cells and to increase HIV-1 transmission to T cells [21], [22], [91], [100]. Moreover, we demonstrate that PPARγ and LXR signaling can interfere with the migration of DCs in response to a CCL21 chemokine gradient (Figure 2A). This appears to be due to the effects of PPARγ and LXR signaling on the expression of CCR7 (Figure 2B), one of the receptors for CCL21. CCR7 is up-regulated upon DC maturation and has been shown to be important for the migration of DCs from the mucosa to regional lymph nodes *in vivo* [54]. By preventing DC migration in response to CCL21, PPARγ and LXR ligands may help to block the dissemination of DC-associated virus from mucosal sites of infection to regional lymph nodes.

Recent studies demonstrated that activation/maturation of DCs through TLR4 or TLR2/TLR1 enhances HIV-1 transmission to target cells via increased HIV-1 capture [23], [24], [25], [92] and Figure 4 and 5). Here, we demonstrate that activating PPARγ or LXR signaling pathways in DCs decreases the ability of both immature and TLR-matured DCs to capture and transfer HIV-1 to T cells (Figure 3, 4A and 5A). Furthermore, NR signaling can inhibit HIV-1 transfer by previously matured DCs (Figure 4C) These results suggest that PPARγ and LXR signaling alter other pathways involved with HIV-1 *trans*-infection that are independent of the maturation state of the DC (Figure 4C), however we cannot rule out the possibility that the prevention of DC maturation may contribute to the NR-mediated decrease in HIV-1 capture and transfer. Many studies have demonstrated a role for PPARγ and LXR signaling in cholesterol metabolism and transport [29], [39], [40]. For example, both signaling pathways stimulate the expression of ABCA1 and ABCG1, which have been implicated in apolipoprotein A1 (ApoA1)- and high density lipoprotein (HDL)-mediated cholesterol efflux, respectively [39]. Given the importance of cholesterol for a number of aspects of HIV-1 biology, including virus binding and infection [42], [43], [44], [45], [47], [48], [49], [50], [51], [76], we hypothesized that PPARγ and LXR signaling was altering the cholesterol content of DC membranes, thereby rendering them incapable of efficiently binding HIV-1 particles. Previous studies have demonstrated that treatment with cholesterol depleting drugs, such as methyl-β-cyclodextrin, or with cholesterol synthesis inhibitors, such as HMGCoA-reductase inhibitors (statins), alters the ability of cells, including DCs, to bind HIV-1 and renders them refractory to HIV-1 infection [42], [43], [45], [49], [50], [51], [101]. Here, we show that cholesterol repletion of PPARγ and LXR ligand-treated DCs reverses the effects of the NR ligands on virus capture and transfer (Figure 9C and 9D), confirming that PPARγ and LXR are mediating their effects through membrane cholesterol. In addition, targeted shRNA knock-down of ABCA1 abrogates the effects of PPARγ and LXR signaling on HIV-1 capture and transfer (Figure9F and 9G). A recent study suggests that LXR-dependent cholesterol efflux in macrophages is mediated entirely through ABCA1, with little to no contribution from ABCG1 [102]. We cannot, however, formally exclude a contribution from ABCG1-dependent cholesterol efflux to the effects we report here. Our data show that PPARγ and LXR signaling decrease cellular cholesterol content, which may in turn deplete cholesterol from membrane lipid rafts. It will be interesting to determine whether treatment of DCs with PPARγ and LXR ligands disrupts lipid rafts and whether this accounts for the decreased ability or NR-treated DCs to capture and transfer HIV-1.

We found that PPARγ and LXR ligand treatments do not alter the levels of a number of known virus attachment factors expressed on DCs including CD4, CCR5, and DC-SIGN (Figure 5F). Moreover, PPARγ and LXR signaling prevents the capture of Env-deficient HIV-1 virus like particles (Figure 8A), suggesting that virus envelope glycoprotein/receptor interactions are not involved in the observed effect. That the effects of PPARγ and LXR signaling on HIV-1 capture are virus envelope glycoprotein-independent is supported by our finding that treatment of DCs with ciglitazone or TO-901317 prevents the capture of viral particles pseudotyped with the envelope glycoproteins of Ebola virus and Marburg virus (Figure 8B). Interestingly, these two viruses are known to require cholesterol for infection [74], [75]. In contrast, treatment with the NR ligands had no effect on the ability of DCs to capture virus particles pseudotyped with the envelope glycoprotein of VSV. Previous studies have demonstrated that VSV-G-pseudotyped HIV-1 particles are efficiently captured by cells depleted of cholesterol using methyl-β-cyclodextrin [43], [75], [76]. These data further support the hypothesis that PPARγ and LXR signaling alter the membrane cholesterol content of DCs, rendering them incapable of efficiently capturing HIV-1 particles.

Although NR ligand treatment limits the expression of immune-activating cytokines and co-stimulatory molecules that are up-regulated as DCs mature, we found that it does not alter the ability of DCs to form conjugates with T cells. The number of DC-T cell conjugates formed with PPARγ and LXR ligand-treated DCs was comparable to that of control untreated DCs (Figure 7A). It will be interesting to determine whether these conjugates represent functional immunological synapses between DCs and T cells. It is worth noting that DC-to-T cell transfer of HIV-1 most likely occurs through the formation of virological synapses [103], [104], [105], [106], [107], [108]. We found that NR ligand treatment does not prevent the formation of virological synapses between DCs and T cells as assessed by confocal microscopy, although ligand treatment does seem to decrease the amount of virus concentrated at the virological synapse (Figure 7B).

Beyond demonstrating the ability of PPARγ and LXR signaling pathways to prevent DC capture and transfer of virus, our results provide support for a number of observations regarding the interactions between DCs and HIV-1. First, we demonstrate that immature DCs can transfer single round replication competent virus to T cells through a Transwell insert that prevents direct contact between the two cell types (Figure 3). Although direct cell-cell contact is required for efficient virus transfer, our data suggest that approximately 20% of infectious virus can be transferred by immature DCs via exosomes or shedding from the cell surface. In contrast, although mature DCs bind approximately 10-fold more virus, less than 10% of transfer is mediated through cell-surface bound viral particles (Figure 4A). Second, our data suggest that a large percentage of virions captured by DCs is internalized or otherwise protected from proteases (Figure 5E). Previous studies have demonstrated that DCs internalize HIV-1, resulting in either degradation of virus particles [56], [65], [109], establishment of productive infection [110], [111], [112], or sequestration into protected intracellular compartments [56], [73], [94], [95]. Although PPARγ and LXR signaling alters the amount of virus captured by DCs, it does not seem to alter the percentage of captured virus that is internalized by DCs (Figure 5E). This is not surprising, since PPARγ and LXR ligand treatment does not alter the endocytic capacity of DCs, as measured by the internalization of FITC-dextran (Figure S2). Finally, our data confirm that DCs can bind virus particles in a gp120-independent manner (Figure 8A). Recent reports demonstrate that host cell-derived GSLs incorporated into the budding virus particle play a critical role in mediating HIV-1 capture by immature and mature DCs in a gp120-independent manner [72], [73]. Taken together with current and previous findings that cholesterol depletion from DC membranes prevents HIV-1 binding [51] (and Figure 9C), these data argue for the presence of a yet-to-be-identified GSL-recognizing attachment factor(s) within lipid raft-like membrane microdomains at the surface of DCs whose function is compromised upon NR ligand treatment.

NR signaling may have beneficial effects on the prevention of HIV-1 transmission beyond the effects on pro-inflammatory cytokine production, migration, and virus capture and transfer. STIs, through engagement of TLRs, and STI/TLR-induced inflammation, can directly activate HIV-1 replication in infected cells. Our data suggest that both PPARγ and LXR ligands repress HIV-1 replication in DCs (Figure 3), although the levels of replication in this cell type are quite low. This finding is consistent with previous studies that have shown that PPARγ ligands repress HIV-1 expression in infected monocytes and macrophages [113], [114], [115]. Recent findings from our laboratory suggest that NR-mediated repression of HIV-1 replication is due to *trans*-repression (T. Hanley and G. Viglianti, manuscript in preparation), as is thought to be the case for NR-mediated repression of pro-inflammatory cytokine production [30], [31], [32], [33], [34], [35], [36], [37], [38]. Although our data suggest that the majority of virus transferred to T cells is due to virus captured by DCs, and not due to virus newly synthesized in infected DCs, NR-mediated inhibition of HIV-1 replication may contribute to the inhibition of *trans*-infection that we report here. By preventing HIV-1 replication, in addition to DC migration, pro-inflammatory cytokine and chemokine production, and *trans*-infection, PPARγ and LXR ligands may block the dissemination of DC-associated virus from the local site of infection to regional lymph nodes.

In the absence of an effective vaccine for HIV-1, the development of topical microbicides that block the early steps of HIV-1 infection and transmission may represent the best option for containing the spread of this global pandemic. To date, there has been limited success with antiviral microbicides. In order to ensure success with future microbicide development, a much greater understanding of the mechanisms involved in the very early stages of mucosal infection and transmission of HIV-1, and the role of DCs in HIV-1 pathogenesis, in particular, are required. Our results contribute to a better delineation of the mechanisms underlying the HIV-1 *trans*-infection activity of DCs, while having implications for the development of new anti-HIV microbicide strategies. PPARγ and LXR ligands are small lipophilic molecules that readily diffuse across cell membranes and might be amenable to topical formulations. Two PPARγ agonists, rosiglitazone and pioglitazone, are currently approved for the systemic treatment of type II diabetes. A limitation of the present study is that we have not yet examined the effects of NR signaling on HIV-1 transmission in the context of a complex tissue model or an animal model. Despite this limitation, the anti-inflammatory and anti-HIV-1 activities of PPARγ and LXR provide a solid rationale for considering them as drug targets that can act synergistically with conventional anti-viral microbicides that target other aspects of mucosal transmission including virion structure, virus binding/entry, or reverse transcription.

## Materials and Methods

### Ethics statement

This research has been determined to be exempt by the Institutional Review Board of the Boston University Medical Center since it does not meet the definition of human subjects research.

### Cell isolation and culture

Primary human CD14+ monocytes were isolated from the peripheral blood mononuclear cells (PBMCs) of healthy donors using anti-CD14 magnetic beads (Miltenyi Biotec) per the manufacturer's instructions. CD14+ monocytes (1.5×10^6^ cells/ml) were cultured in RPMI 1640 supplemented with 10% FBS, 100 U/ml penicillin, 100 µg/ml streptomycin, 0.29 mg/ml L-glutamine, 1000 U/ml IL-4 (PeproTech), and 1400 U/ml GM-CSF (PeproTech) for 6-8 days at the end of which the cells acquired an immature dendritic cell phenotype as assessed by flow cytometry (CD11c^+^, DC-SIGN^+^, HLA-DR^lo^, CD80^−^, CD86^−^). Cells were given fresh medium supplemented with IL-4 and GM-CSF every 2 days. Mature dendritic cells were obtained following 48 hour exposure to 100 ng/ml ultra-pure *E. coli* K12 LPS or 100 ng/ml PAM3CSK4. Primary human myeloid DCs (mDCs) and plasmacytoid DCs (pDCs) were isolated from monocyte- and B cell-depleted PBMCs using anti-CD11c and anti-BDCA4 magnetic beads (Miltenyi Biotec) per the manufacturer's instructions. mDCs were cultured in RPMI 1640 with 10% FBS, 100 U/ml penicillin, 100 µg/ml streptomycin, 0.29 mg/ml L-glutamine, 1000 U/ml IL-4, and 1400 U/ml GM-CSF. pDCs were cultured in RPMI 1640 supplemented with 10% FBS, 100 U/ml penicillin, 100 µg/ml streptomycin, 0.29 mg/ml L-glutamine, and 10 ng/ml IL-3 (PeproTech). Primary human CD4+ T cells were isolated from CD14-depleted peripheral blood mononuclear cells using anti-CD4 magnetic beads (Miltenyi Biotec) per the manufacturer's instructions. CD4+ T cells (2×10^6^ cells/ml) were cultured in RPMI 1640 supplemented with 10% FBS, 100 U/ml penicillin, 100 µg/ml streptomycin, 0.29 mg/ml L-glutamine, 50 U/ml IL-2 (R&D Systems), and 5 µg/ml PHA-P (Sigma) for 6-8 days at the end of which the cells acquired a memory T cell phenotype as assessed by flow cytometry (CD3^+^, CD4^+^, CD45RO^+^, CD45RA^–^). 293T cells were cultured in DMEM supplemented with 10% FBS, 100 U/ml penicillin, 100 µg/ml streptomycin, and 0.29 mg/ml L-glutamine. MAGI-CCR5 cells were cultured in DMEM supplemented with 10% FBS, 100 U/ml penicillin, 100 µg/ml streptomycin, 0.29 mg/ml L-glutamine, 500 µg/ml G418, 1 µg/ml puromycin, and 0.1 µg/ml hygromycin B. PM1 cells were cultured in RPMI 1640 supplemented with 10% FBS, 100 U/ml penicillin, 100 µg/ml streptomycin, and 0.29 mg/ml L-glutamine.

### Nuclear receptor and Toll-like receptor ligands

The LXR ligand TO-901317 was purchased from Calbiochem. The PPARγ ligands ciglitazone and rosiglitazone were purchased from Cayman Chemicals. The ligands were reconstituted in DMSO. The TLR2 ligand PAM3CSK4, the TLR4 ligand *E. coli* K12 LPS, the TLR7 ligand CLO97, and the TLR9 ligand CpG ODN 2006 were purchased from Invivogen. Unless otherwise noted, DCs were treated with PPARγ and LXR ligands for 24–48 hours, beginning one hour prior to treatment with TLR ligands.

### Virus production

Replication competent R5-tropic HIV-1_ADA_ and X4-tropic HIV-1_NL4-3_ were generated by infection of PM1 cells. Single-round replication-competent HIV-1-based reporter viruses were generated by packaging a luciferase expressing reporter virus, BruΔEnvLuc2, with the envelope glycoproteins from CCR5-tropic HIV-1(Ada-M), CXCR4-tropic HIV-1(HXB2), VSV (VSV-G), Ebola virus Zaire (EboV-Z), Ebola virus Sudan (EboV-S), or Marburg virus (MarV). EGFP-labeled virus particles were generated by co-transfection of the pro-viral clone HIV-1_NL4-3_ with an expression vector encoding a Vpr-EGFP fusion protein. Virus stocks were generated by transfecting HEK293T cells using the calcium phosphate method. All viruses were titered on MAGI-CCR5 cells and p24^gag^ content was determined by ELISA.

### Transfer assays

To assess DC-mediated transfer of HIV-1 to T cells, DCs were incubated with Ada-M- or HXB2-pseudotyped HIV-luciferase reporter virus at an MOI  = 0.1 (37.8–40.4 ng p24^gag^) for four hours at 37°C. Cells were washed five times with PBS to remove unbound virus, seeded in 96-well plates (2.5×10^5^ cells/well), and then cultured with either PM1 T cells (5×10^5^ cells/well) or autologous primary CD4+ T cells (5×10^5^ cells/well) for 48 hours. In some instances, the DCs were seeded in 24-well plates separated from the T cells by a Transwell insert (Corning) with a 0.4 µm pore size. As controls, virus-exposed DCs and virus-exposed T cells were cultured alone for 48 hours. After 48 hours, the cells were harvested, washed two times with PBS, and lysed in PBS/0.02% Triton X-100. Protein levels in cell lysates were determined using a modified Lowry protein assay (BioRad) and luciferase activity was measured using luciferase reagent (Promega) and a MSII luminometer (Molecular Devices). In some experiments, replication competent R5-tropic HIV-1_ADA_ or X4-tropic HIV-1_NL4-3_ (5 ng p24^gag^) were used in place of packaged reporter virus and transfer was measured by p24^gag^ ELISA.

### Capture assays

DCs (2.5×10^5^ cells/well) were incubated with replication competent R5-tropic HIV-1_ADA_ or X4-tropic HIV-1_NL4-3_ (5 ng p24^gag^) for three to four hours at 37°C. Cells were washed four to five times with PBS to remove unbound virus, and lysed in PBS/10%FBS/0.5% Triton X-100. In some experiments, virus-exposed DCs were incubated with 0.5% trypsin for 5 minutes at 37°C to degrade surface-bound virus particles, washed twice in culture medium, and then lysed as above. An ELISA was used to determine the amount of p24^gag^ protein associated with the cells. In some experiments, Ada-M-, HXB2-, VSV-G, EboV-, or MarV-packaged HIV-luciferase reporter virus (5 ng p24^gag^) or an equal amount of reporter virus lacking envelope glycoproteins (ΔEnv) was used.

### DC-T cell conjugate formation assays

Primary CD4+ T cells were labeled with the cytoplasmic dye CMTMR (CellTracker Orange, Molecular Probes) for 30 minutes at 37°C and then washed three times with PBS to remove excess dye. Cells were then incubated for 16 hours at 37°C and washed twice with PBS prior to use in conjugate formation assays. Following labeling, 5×10^5^ T cells were incubated with 2.5×10^5^ unlabeled iMDDCs for four hours at 37°C in a total volume of 200 µl. The conjugates were then fixed by gently adding an equal volume of 4% paraformaldehyde. Samples were run immediately through a flow cytometer. Conjugate formation was assessed by fluorescence associated with the MDDC population.

### Virological synapse formation assays

Primary CD4+ T cells were labeled with the cytoplasmic dye CMCA (CellTracker Blue, Molecular Probes) for 30 minutes at 37°C and then washed three times with PBS to remove excess dye. T cells were then incubated for 16 hours at 37°C and washed twice with PBS prior to use in virological synapse formation assays. 2.5×10^5^ unlabeled mMDDCs were incubated with 100 ng HIV-1_NL4-3_ virions packaged with Vpr-EGFP for four hours at 37°C, washed four times with PBS, and incubated with 5×10^5^ CMCA-labeled autologous T cells for four additional hours. The cells were fixed in 1% paraformaldehyde, stained with anti-CD81-PE (BD Pharmingen). Z-stacks were captured on the Nikon deconvolution wide-field Epifluorescence Scope at 100×. Using ImageJ software, the images were deconvolved and the fluorescence was summed.

### Cholesterol repletion assays

Cholesterol-saturated methyl-β-cyclodextrin was prepared as previously described [116]. Briefly, cholesterol powder was added to 240 mM methyl-β-cyclodextrin solution at 1.16 mg/ml, agitated overnight, and filter sterilized using a 0.22-µm filter. To replete cholesterol, MDDCs were incubated with cholesterol-saturated methyl-β-cyclodextrin at a concentration of 300 µM cholesterol for 30 minutes at 37°C and then washed five times with PBS before being used in virus capture and transfer studies.

### Transwell migration assays

MDDCs (2.5×10^5^ cells) were seeded above a Transwell insert with a 5 µm pore size and allowed to migrate through the insert in response to medium or CCL21 (PeproTech). Cells above and below the Transwell insert were fixed in 2% paraformaldehyde and counted in a hemocytometer to determine the relative migratory capacity of the MDDCs. Migration index was calculated by dividing the number of experimental cells that migrated in response to CCL21 by the number of untreated cells that migrated in response to media alone.

### Cholesterol efflux assays

Cholesterol efflux into cell-free culture supernatants and cholesterol content of lysed MDDCs were measured using the AmplexRed cholesterol assay kit per the manufacturer's instructions (Invitrogen).

### shRNA knock-down of ABCA1

MDDCs were transfected with plasmids that encoded either a mixture of three to five shRNAs directed against ABCA1 or a mixture of control shRNAs (Santa Cruz Biotechnology) and a puromycin-resistance gene using Oligofectamine (Invitrogen) per the manufacturer's instructions. Transfected cells were selected by culture in the presence of puromycin for 48 hours and then used for cholesterol efflux assays, used for HIV-1 capture assays, or lysed for immunoblot analysis to measure ABCA1 expression.

### Flow cytometry

MDDC phenotypes were assessed using antibodies against HLA-DR, CD80, CD86, DC-SIGN, CD11c, CD4, CCR5, CXCR4, and CCR7. Primary CD4+ T cell phenotypes were assessed using antibodies to CD3, CD4, CD8, CD45RO, CD45RA, CCR5, and CXCR4. Flow cytometric data was acquired using a Becton-Dickenson FACScan II and data was analyzed using FlowJo software.

### Cytokine and chemokine release assays

MDDCs (2.5×10^5^ cells/well) or pDCs (1×10^5^ cells/well) were treated with PAM3CSK4 (100 ng/ml), LPS (100 ng/ml), CLO97 (1 µg/ml), or CpG ODN 2006 (5 µM) for 24 hours in the presence or absence of nuclear receptor ligands as described in the legend to figure 1. Cell-free culture supernatants were collected and analyzed for TNF-α (eBioscience), IL-6 (eBioscience), IL-8 (BioLegend), MIP-1α (PeproTech), and RANTES (PeproTech) release by commercially-available ELISA following the manufacturer's instructions.

### Cell viability assays

MDDC cell viability was assessed by trypan blue dye exclusion, MTT cytotoxicity assay, and LDH release using a commercial kit (Promega) per the manufacturer's instructions.

### Statistical analysis

Untreated control and ligand-treated experimental samples were compared using a two-tailed t-test. Experiments were performed in duplicate (mDCs and pDCs) or triplicate (MDDCs) using cells from a minimum of three different donors as indicated in the figure legends (n). Data are presented as the mean ± standard deviation of pooled data from at least three donors.

### Accession numbers

PPARγ Swiss-Prot # P37231; LXRα Swiss-Prot # Q13133; LXRβ Swiss-Prot # P55055; CCR7 Swiss-Prot # P32248; CCL21 Swiss-Prot # O00585;TLR1 Swiss-Prot # Q15399; TLR2 Swiss-Prot # O60603; TLR4 Swiss-Prot # O00206; TLR7 Swiss-Prot # Q9NYK1; TLR9 Swiss-Prot # C3W5P5; ABCA1 Swiss-Prot # O95477; ABCG1 Swiss-Prot # P45844.

## Supporting Information

Figure S1 PPARγ and LXR ligand treatment do not alter MDDC viability. Immature MDDCs were treated for 48 hours with 100 µM ciglitazone or 1 µM TO-901317. Cell viability was assessed by the release of LDH into the cell culture supernatant. (n = 3).
(0.09 MB TIF)

Figure S2 PPARγ and LXR ligand treatment inhibit HIV-1 binding to MDDCs. Immature MDDCs were treated for 48 hours with 100 µM ciglitazone or 1 µM TO-901317 and incubated with HIV-1_ADA_ for 3–4 hours at 4°C. Following incubation with virus, cells were washed four times with ice cold PBS and then lysed. Virus capture was measured by p24^gag^ ELISA. (n = 3) * p<0.001 compared to untreated controls.
(0.05 MB TIF)

Figure S3 PPARγ and LXR ligand treatment do not prevent MDDC uptake of FITC-dextran. Immature MDDCs treated with 100 µM ciglitazone (upper panel) or 1 µM TO-901317 (lower panel) were incubated with FITC-dextran (100 µg/ml) for two hours at 37°C and then washed extensively to remove unbound FITC-dextran. The cells were then fixed in 2% paraformaldehyde and uptake of FITC-dextran was assessed by flow cytometry. Shaded histogram, MDDCs without FITC-dextran. Blue line, untreated MDDCs. Red line, nuclear receptor ligand-treated MDDCs.
(0.32 MB TIF)
