# Supplementary figures and images for: PPARγ and LXR Signaling Inhibit Dendritic Cell-Mediated HIV-1 Capture and trans-Infection
Source: PLoS Pathog. 2010 Jul 1;6(7):e1000981. doi: 10.1371/journal.ppat.1000981 (PMC2895661; doi:10.1371/journal.ppat.1000981)

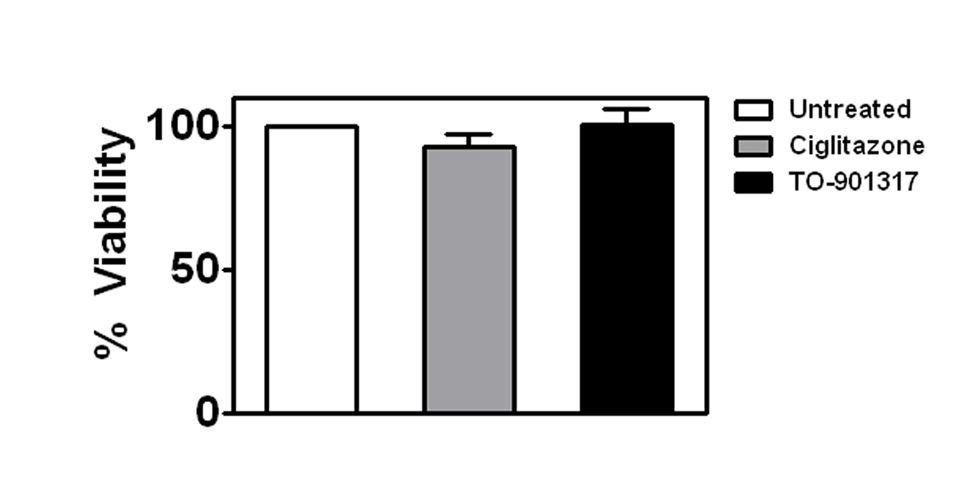

Supplement: Figure S1 — PPARγ and LXR ligand treatment do not alter MDDC viability. Immature MDDCs were treated for 48 hours with 100 µM ciglitazone or 1 µM TO-901317. Cell viability was assessed by the release of LDH into the cell culture supernatant. (n = 3). (0.09 MB TIF) [file ppat.1000981.s001.tif]

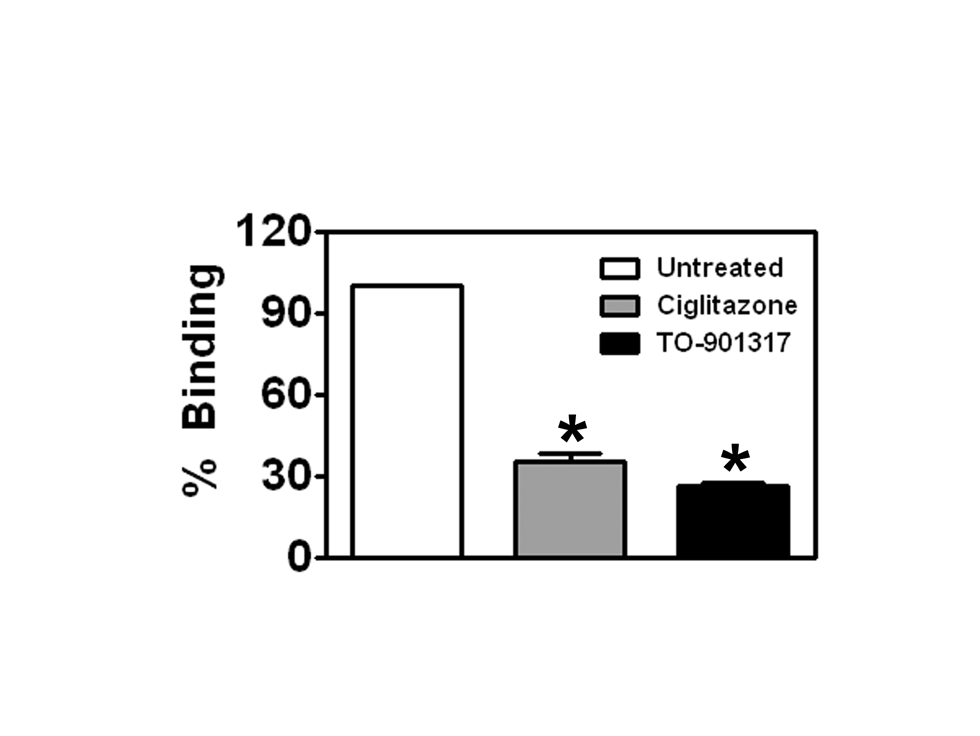

Supplement: Figure S2 — PPARγ and LXR ligand treatment inhibit HIV-1 binding to MDDCs. Immature MDDCs were treated for 48 hours with 100 µM ciglitazone or 1 µM TO-901317 and incubated with HIV-1ADA for 3–4 hours at 4°C. Following incubation with virus, cells were washed four times with ice cold PBS and then lysed. Virus capture was measured by p24gag ELISA. (n = 3) * p<0.001 compared to untreated controls. (0.05 MB TIF) [file ppat.1000981.s002.tif]

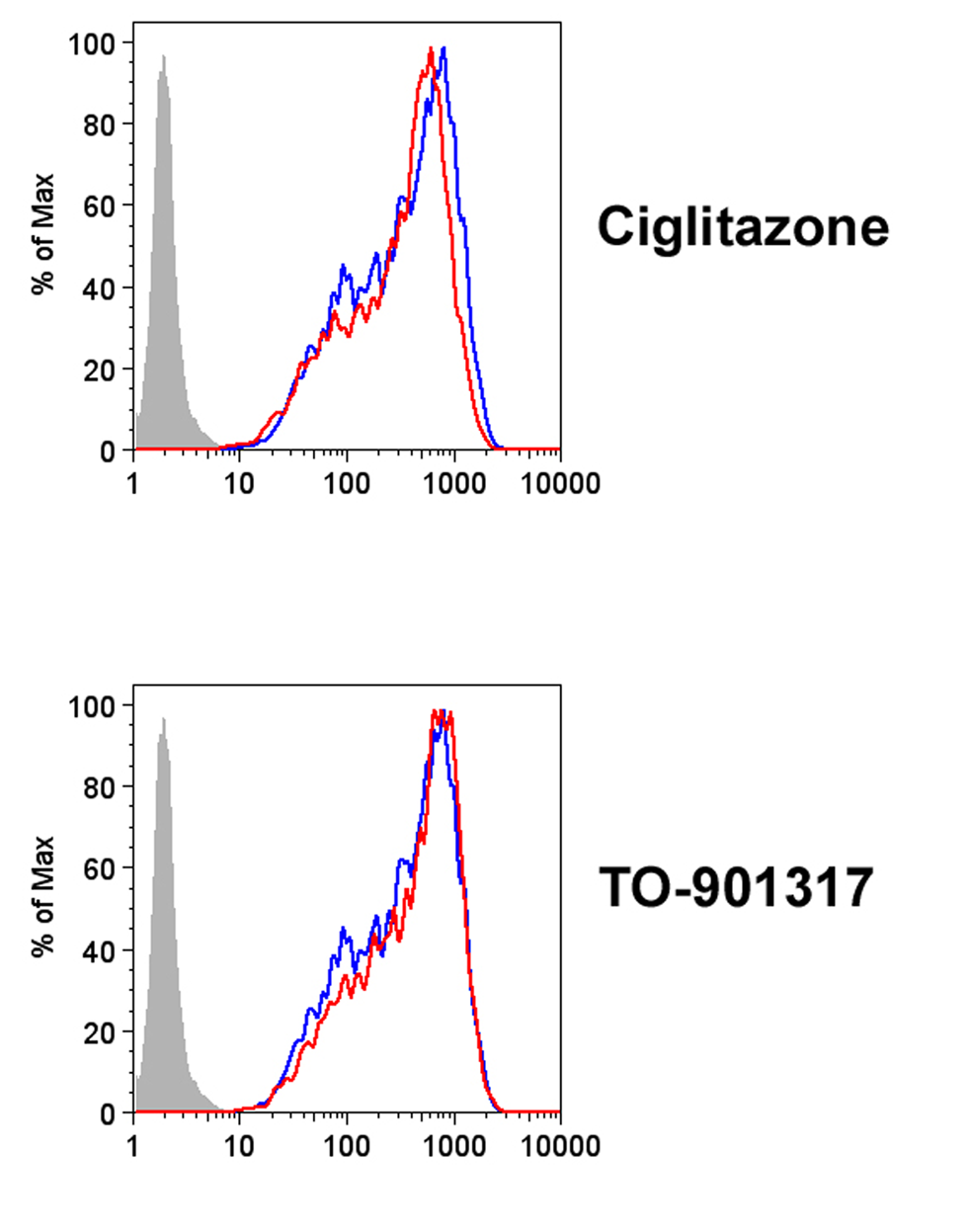

Supplement: Figure S3 — PPARγ and LXR ligand treatment do not prevent MDDC uptake of FITC-dextran. Immature MDDCs treated with 100 µM ciglitazone (upper panel) or 1 µM TO-901317 (lower panel) were incubated with FITC-dextran (100 µg/ml) for two hours at 37°C and then washed extensively to remove unbound FITC-dextran. The cells were then fixed in 2% paraformaldehyde and uptake of FITC-dextran was assessed by flow cytometry. Shaded histogram, MDDCs without FITC-dextran. Blue line, untreated MDDCs. Red line, nuclear receptor ligand-treated MDDCs. (0.32 MB TIF) [file ppat.1000981.s003.tif]
